# Public Dental Expenditure and Dental Visits in Europe and Japan

**DOI:** 10.1111/cdoe.70076

**Published:** 2026-05-31

**Authors:** Yuko Inoue, Jun Aida, Richard G. Watt, Anja Heilmann

**Affiliations:** ^1^ Department of Clinical Epidemiology and Health Economics The University of Tokyo Tokyo Japan; ^2^ Department of Dental Public Health Institute of Science Tokyo Japan; ^3^ Department of Epidemiology and Public Health University College London London UK

**Keywords:** dental public health, epidemiology, health services research, inequalities, public health

## Abstract

**Background:**

Dental care is important for oral health, but access to dental services is subject to social inequalities and varies between countries. Despite this, few cross‐national studies have examined how public dental expenditure relates to dental service use among older adults. This study aimed to investigate the associations between public dental expenditure and dental visits among older adults using an international comparative design.

**Methods:**

Data were obtained from the Survey of Health, Ageing and Retirement in Europe (SHARE, Wave 8, 2019), covering 25 European countries and Israel, and the Japanese Gerontological Evaluation Study (JAGES, 2019). The final sample included 48 497 adults aged 65 years or older. The outcome was any dental visit in the past year, while the explanatory variable was country‐level annual per capita public dental expenditure on outpatient dental care in 2019 (USD), obtained from the OECD database and adjusted for purchasing power parity. A multilevel modified Poisson regression model was used to estimate prevalence ratios (PRs), accounting for between‐country variability. Interaction terms between household income and public dental expenditure were included to examine effect modification.

**Results:**

The overall dental visit rate was 59.5%, with higher rates among women, those with higher education, and those in higher income groups. Each $10 increase in per capita public dental expenditure at the country level was associated with higher dental visit rates (PR: 1.05, 95% CI: 1.03–1.07). The association was stronger among lower‐income groups, suggesting that income‐related inequalities in dental visits were smaller in countries with higher public dental expenditure.

**Conclusions:**

Public expenditure on dental care was associated with higher dental visit rates, particularly among low‐income groups. These findings suggest that public financing of dental care may help reduce income‐related inequalities in dental visits among older adults.

## 1 Introduction

Oral diseases affect an estimated 3.7 billion people worldwide, representing nearly half the world's population [1, 2]. Untreated dental caries in permanent teeth is the most common chronic condition, with a global prevalence of 27.5%, while severe periodontal disease affects around 12.5% of all people [1]. These conditions impair quality of life and are associated with systemic diseases such as cardiovascular disease, diabetes [3], and pneumonia [4]. Dental care contributes to the prevention and management of oral diseases and may have implications for general health, as suggested by recent studies [5, 6].

While most oral diseases are preventable and treatable in their early stages, dental care is often inadequately integrated into universal health coverage (UHC) systems [2]. Consequently, patients in many countries face substantial out‐of‐pocket expenses [7, 8, 9]. Even in high‐income countries, public reimbursement for dental care is often insufficient, leading to continued reliance on private insurance and direct payments [10, 11]. In contrast, some nations have expanded public dental coverage to mitigate these financial barriers. Notably, Japan and Germany provide comprehensive dental services under statutory health insurance (SHI). Among OECD countries, only Japan, Germany, and Slovakia cover over half of adult dental treatment costs [12]. Japan's particularly low out‐of‐pocket costs [13] and high dental care utilisation rates [10] make it a valuable case for comparative research. Understanding the role of public dental expenditure in lowering financial barriers is essential to assessing its impact on access and equity in oral health care.

Previous studies have reported income‐related inequalities in dental service utilisation, reflecting persistent social disparities in access to oral healthcare [14]. A previous European study also examined whether country‐level public expenditure on health and healthcare system characteristics were associated with dental check‐up use [15]. However, few cross‐national studies have quantitatively assessed the extent to which public dental expenditure may attenuate these inequalities. Previous studies have examined socioeconomic inequalities and patterns of dental service use in relation to public coverage [16] and welfare state regimes [17], but analyses directly quantifying national‐level public dental expenditure remain limited. Accordingly, this study aimed to investigate the associations between public dental expenditure and dental visits among older adults using data from 25 European countries, Israel, and Japan, and to examine whether these associations differed by household income.

## 2 Methods

### 2.1 Study Design and Data

This study employed a cross‐sectional design using data from two large‐scale cohort studies: Wave 8 (2019) of the SHARE and the 2019 JAGES. SHARE is a multinational longitudinal survey examining ageing‐related factors across 25 European countries and Israel [18]. The eighth wave, used in this study, collected responses from 46 733 individuals aged 50 and older across these countries. In SHARE Wave 8, individual retention rates for the longitudinal sample ranged from approximately 60% to 74% across countries [19]. The JAGES project is an ongoing prospective cohort study of individuals aged ≥ 65 years [20]. In 2019, the JAGES survey covered 192 484 individuals, of whom 24 353 responded to the questionnaire that included items on dental care, yielding a response rate of 53.0% among those who received the dental questionnaire.

The SHARE study received ethical approval from the Ethics Council of the Max Planck Society and national ethics committees in participating countries. The JAGES 2019 survey received ethical approval from the Ethics Committee of the National Centre for Geriatrics and Gerontology (Approval No. 1274–2) and Chiba University (Approval No. 3442). All participants provided informed consent before participation.

This study was conducted and reported in accordance with the STROBE and OHStat guidelines for observational research.

### 2.2 Analytical Sample

Exclusion criteria were applied based on participant characteristics and data completeness. In the SHARE, participants younger than 65 years (*n* = 13 953), those residing in long‐term care facilities (*n* = 485), and those with missing responses for the primary outcome variable, dental visits (*n* = 149), were excluded. Similarly, in the JAGES, participants who reported currently requiring long‐term care (*n* = 2 811), those with missing responses for dental visits (*n* = 1 192), those with missing equivalised income data (*n* = 2 489), and those with missing regional data required for calibration (*n* = 3) were excluded. Figure 1 shows the flow of participant selection in this study.

**FIGURE 1:**
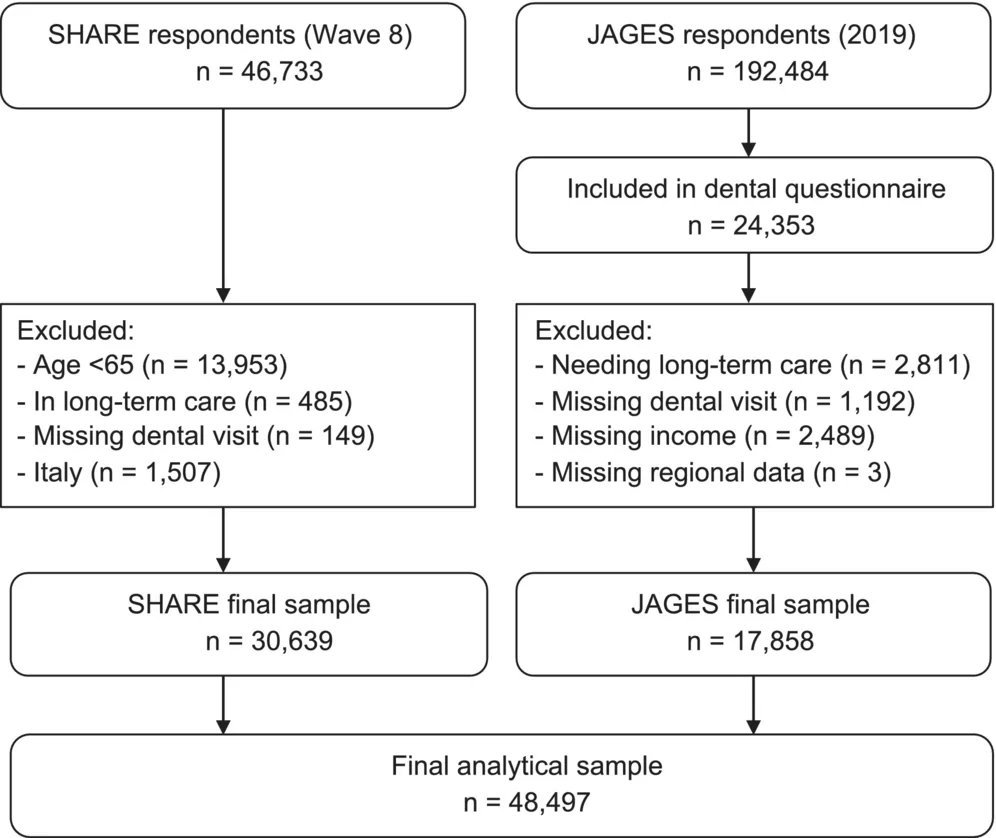
Flow diagram of participant selection from SHARE (2019) and JAGES (2019) datasets.

In SHARE, missing income data are imputed using a robust method incorporating spouse‐reported income, assets, and expenditures, ensuring high‐quality data [19]. These imputed income values were used as provided and treated as complete in the analysis, without further imputation. In contrast, JAGES does not include similarly imputed income variables. Although missing income data in JAGES were initially considered for imputation, the imputation models failed to converge due to substantial differences between participants with and without income data. Therefore, participants with missing income data in JAGES were excluded from the analysis. Italy was excluded entirely from the analysis because national‐level data on public dental expenditure were unavailable. For all other variables with missing data, multiple imputation by chained equations (MICE) was performed, creating 20 imputed datasets.

### 2.3 Outcome

To ensure comparability between SHARE and JAGES, dental visits were defined as having occurred within the past year, regardless of the purpose of the visit. This harmonisation was necessary because the SHARE survey did not distinguish between treatment and preventive care. The primary outcome variable was dental visits in the past year. In SHARE, participants were asked, “During the last twelve months, have you seen a dentist or a dental hygienist?” Responses of “Yes” were coded as 1 (visited), and responses of “No” were coded as 0 (did not visit). In JAGES, two questions assessed dental visits: “When was the last time you visited a dental clinic for treatment (including denture adjustments)?” and “When was the last time you visited a dental clinic for purposes other than treatment (e.g., check‐ups)?” Responses indicating visits “within six months” or “six months to a year ago” to either question were coded as 1 (visited), while all other responses were coded as 0 (did not visit).

### 2.4 Explanatory Variable

The key explanatory variable was public dental expenditure per capita in 2019, obtained from OECD Health Expenditure and Financing data [21], adjusted for purchasing power parity and expressed in 10 USD increments. This expenditure represents outpatient dental curative care and includes government schemes, social health insurance schemes, and compulsory private insurance schemes.

### 2.5 Covariates

A range of covariates at the individual and country levels was included based on previous literature. Demographic factors included sex (male, female), age group (65–74, 75–84, and 85+ years), and marital status (partnered, unpartnered). Socio‐economic position was assessed using education, household income, and employment status, as these were the most comparable socioeconomic indicators available across SHARE and JAGES for harmonised analysis. Educational attainment was standardised according to the ISCED 1997 classification. In JAGES, educational levels were recoded to match SHARE categories: Primary education (levels 0–2), secondary education (level 3), and post‐secondary education, including vocational and higher education (levels 4–8). Equivalised household income was calculated by dividing total household income by the square root of the household size. To harmonise JAGES income data with SHARE, income categories were converted into continuous Euro‐equivalent values adjusted for purchasing power parity (PPP). To account for income distribution differences across countries, participants were grouped into four income categories based on the 25th, 50th, and 75th percentiles of equivalised income within each country. Individuals were then classified into four income groups: Low, middle‐low, middle‐high, and high. Employment status was categorised as retired, employed, or unemployed/other.

Self‐rated health was categorised as very good, good, fair, or poor. To ensure comparability between datasets, SHARE's five‐point scale was recoded by combining “Excellent” and “Very good” into a single “Very good” category, with the remaining levels matched accordingly to JAGES responses.

Dental pain was assessed as a binary variable. In SHARE, respondents who answered “yes” to the question “Are you troubled with pain?” and additionally reported pain in the “mouth/teeth” area were classified as having dental pain. In JAGES, respondents were asked, “Have you experienced tooth pain in the past six months?” Responses of “None at all” were categorised as no dental pain, while all other options (“Hardly any,” “Occasional,” “Frequent,” and “Constant”) were classified as dental pain present.

Smoking status was categorised as current smoker or not currently smoking. In SHARE, the question “Do you smoke at the present time?” was used to classify respondents. In JAGES, smoking status was based on the response to “Do you currently smoke?” Respondents who answered “Almost every day” or “Occasionally” were classified as current smokers, while those who had quit within the past 5 years, quit more than 5 years ago, or never smoked were categorised as not currently smoking.

The number of dentists per 10 000 population was used as a country‐level covariate. These data were obtained from the World Health Organization [22] for the year 2019 to align with the survey timeframe. For Luxembourg (2017) and Japan (2018), where 2019 data were unavailable, the most recent available data were used to ensure consistency across countries.

### 2.6 Statistical Analysis

To account for differences in sample size and to enhance representativeness, calibration weights were applied [23, 24]. The weighting approach followed SHARE's methodology, using the sreweight command in Stata [25], adjusting for age, sex, and geographic regions (NUTS1 for SHARE, prefecture‐level for JAGES). To align JAGES with SHARE, the same calibration method was applied. The calibration weights were then log‐transformed, normalised to a 0–1 scale within each country, and adjusted to prevent zero values by replacing them with a small constant. Advice on the weighting approach was obtained from the SHARE research team prior to weighting.

Following the merging of data from SHARE and JAGES, missing values were handled using multiple imputation by chained equations (MICE). Missing values were imputed based on appropriate regression models, considering whether each variable was categorical or continuous. The variable with the highest proportion of missing values was employment status, with a missing rate of 1.52%. Following standard recommendations [26], 20 imputations were performed to ensure stability of results.

Given the relatively high prevalence of dental visits (59%), a modified Poisson approach was used instead of logistic regression to accurately estimate prevalence ratios, following established methods [27]. Prevalence ratios (PRs) for dental visits were estimated using multilevel Poisson regression with robust standard errors. Models accounted for the hierarchical structure of the data, with individuals (level 1) nested within countries (level 2), adjusting for both individual‐ and country‐level covariates.

To quantify the extent of between‐country variation, the Variance Partition Coefficient (VPC) and the Median Rate Ratio (MRR) were calculated. VPC represents the proportion of total variation attributable to country‐level differences, providing a relative measure of variance decomposition across levels. In contrast, MRR provides an interpretable measure on the rate ratio scale, quantifying the median relative difference in outcome rates between two randomly selected countries. Values above 1.0 indicate meaningful contextual heterogeneity. The MRR shares the same conceptual framework as the median odds ratio (MOR) used in multilevel logistic regression and applies it to Poisson models [28, 29].

To assess the association between public dental expenditure and dental visits, and its potential variation by income level, three sequential models were applied to the multiply imputed and weighted data. All models were estimated using modified Poisson regression with robust standard errors, accounting for the hierarchical structure of individuals nested within countries. Model 1 included individual‐level covariates (age, sex, education, employment status, marital status, self‐rated health, dental pain, smoking status, and income group), along with the number of dentists per 10 000 population at the country level. Model 2 added public dental expenditure as a country‐level covariate. Model 3 introduced interaction terms between public dental expenditure and income group to examine potential effect modification. Predictive margins plots were generated to visualise the association between public dental expenditure and dental visit rates across income groups.

All statistical analyses were conducted using Stata 17.0 [30]. A two‐sided *p*‐value < 0.05 was considered statistically significant.

### 2.7 Sensitivity Analyses

Sensitivity analyses were conducted to assess the robustness of the findings. First, an unweighted version of Model 3 (Model 3A) was tested to examine whether the application of weights influenced the results. Next, Model 3B was estimated excluding participants from Japan, which accounted for 40% of the total sample, to determine whether the overall findings were driven by the inclusion of Japanese data. Lastly, Model 3C was tested by excluding expenditures from compulsory private insurance to assess whether the main results were influenced by variations in insurance coverage. In addition, a supplementary country‐level ecological analysis was conducted to examine the association between public dental expenditure and dental visit rates across the 27 countries (Figure S1, Supporting Information).

## 3 Results

The final analytical sample included 48 497 individuals, comprising 17 858 from JAGES and 30 639 from SHARE. The mean age of participants was 74.4 years (SD = 6.7), and 46.5% were male.

Table 1 summarises dental visits in the past year by participant characteristics and country using imputed data. Dental visits were more common among females, younger age groups, those on higher incomes, and those with higher levels of education. Participants with better self‐rated health, married individuals, and those experiencing tooth pain also reported higher visit rates. Country‐level differences were notable, with Denmark and Sweden showing the highest rates, and Romania and Hungary the lowest.

**TABLE 1:** Sample characteristics by dental visit in the past year among older adults in SHARE Wave 8 (2019) and JAGES 2019 (imputed data; *N* = 48 497).

	Total *N* = 48 497	Not Visited *n* = 19 649	Visited *n* = 28 848
	*n*	%	%
		40.5	59.5
Age (years)			
65–74	26 834	37.2	62.8
75–84	17 435	41.4	58.6
≥ 85	4 228	57.8	42.2
Sex			
Male	22 555	41.5	58.5
Female	25 942	39.7	60.4
Education status			
Primary education	15 304	54.1	45.9
Secondary education	18 721	38.1	61.9
Post‐secondary education	14 472	29.3	70.7
Marital status			
Unmarried	15 122	47.2	52.8
Married	33 375	37.5	62.5
Self‐rated health			
Very good	8 395	29.9	70.1
Good	24 661	35.9	64.1
Fair	11 593	50.0	50.1
Poor	3 848	64.9	35.1
Employment status			
Retired	32 789	43.2	56.8
Employed	12 232	30.0	70.0
Unemployed/Others	3 477	52.0	48.0
Income groups			
Lower Quartile	13 458	47.1	52.9
Middle‐Lower Quartile	11 255	43.1	56.9
Middle‐Upper Quartile	12 439	37.4	62.6
Upper Quartile	11 345	33.6	66.4
Tooth pain			
No	43 237	42.7	57.3
Yes	5 260	22.9	77.1
Current smoking status			
No	34 495	39.2	60.8
Yes	14 002	43.7	56.3
Country			
Austria	1 198	38.5	61.5
Belgium	1 283	34.4	65.6
Bulgaria	577	77.8	22.2
Croatia	730	69.6	30.4
Cyprus	418	63.2	36.8
Czech Republic	2 116	34.3	65.7
Denmark	1 395	14.5	85.5
Estonia	2 209	63.4	36.6
Finland	730	29.9	70.1
France	1 729	44.1	55.9
Germany	1 937	15.8	84.2
Greece	2 006	72.3	27.7
Hungary	579	83.1	16.9
Israel	765	55.6	44.4
Japan	17 858	30.8	69.3
Latvia	476	72.1	27.9
Lithuania	815	61.2	38.8
Luxembourg	556	24.6	75.4
Malta	496	61.9	38.1
Netherlands	1 468	26.4	73.6
Poland	1 264	74.8	25.2
Romania	712	87.6	12.4
Slovakia	406	51.5	48.5
Slovenia	1 773	58.0	42.0
Spain	1 651	61.7	38.3
Sweden	1 967	14.6	85.4
Switzerland	1 383	20.0	80.0

Table 2 presents the annual per capita public dental expenditure on dental curative care in 2019, adjusted for PPP. Germany (262.4 USD) and Japan (180.3 USD) reported the highest expenditures, whereas Greece (0.3 USD) and Spain (1.8 USD) exhibited the lowest levels of expenditure.

**TABLE 2:** Annual per capita public dental expenditure, total expenditure and expenditure by scheme type (2019, PPP‐adjusted USD).

Country	Total Public Dental Expenditure	Breakdown by Scheme Type		
		Government	Social insurance	Compulsory private
Austria	142.4	7.8	134.6	0
Belgium	57.6	0.3	57.3	0
Bulgaria	33.0	< 0.01	33.0	0
Croatia	78.6	0.4	78.2	0
Cyprus	7.2	7.2	0	0
Czechia	72.0	0.03	71.9	0
Denmark	88.4	88.4	0	0
Estonia	66.2	0.1	66.2	0
Finland	62.9	50.6	12.3	0
France	127.8	9.8	71.3	46.7
Germany	262.4	23.7	200.0	38.6
Greece	0.3	0.3	0.01	0
Hungary	27.3	1.4	25.9	0
Israel	3.2	3.2	0	0
Japan	180.3	11.8	168.5	0
Latvia	20.0	20.0	0	0
Lithuania	34.9	1.1	33.8	0
Luxembourg	122.2	0.1	122.2	0
Malta	13.5	13.5	0	0
Netherlands	20.1	0.6	4.8	14.7
Poland	24.5	0.2	24.3	0
Romania	3.1	0	3.1	0
Slovakia	57.2	0.5	56.7	0
Slovenia	71.6	0.2	71.4	0
Spain	1.8	1.1	0.7	0
Sweden	124.5	124.5	0	0
Switzerland	27.7	10.1	8.4	9.2

*Note:* Annual per capita expenditure on outpatient dental care from government and insurance schemes (2019 USD, adjusted for PPP). Data are from the OECD (2019). In the analysis, public dental expenditure was modelled in increments of 10 USD per capita.

Table 3 summarises the multilevel modified Poisson regression models. In the fully adjusted analysis model (Model 3), which included public expenditure and interaction terms, public dental expenditure was significantly associated with a higher prevalence of dental visits (PR = 1.05; 95% CI: 1.03–1.07), indicating a 5% increase in the probability of dental visits for every $10 per capita increase in public spending.

**TABLE 3:** Prevalence Ratios (PR) from multilevel modified Poisson regression models assessing the association between public dental expenditure and dental visits among older adults in SHARE Wave 8 (2019) and JAGES 2019 (*N* = 48 497).

	Null Model	Univariate analysis	Model 1	Model 2	Model 3
	PR (95% CI)	PR (95% CI)	PR (95% CI)	PR (95% CI)	PR (95% CI)
Public dental expenditure (unit = 10 USD per capita)		1.05 (1.03, 1.07)		1.04 (1.02, 1.06)	1.05 (1.03, 1.07)
Age (years)					
65–74		1.00	1.00	1.00	1.00
75–84		0.91 (0.81, 1.02)	0.96 (0.87, 1.05)	0.96 (0.87, 1.05)	0.96 (0.87, 1.05)
≥ 85		0.70 (0.57, 0.86)	0.78 (0.67, 0.91)	0.78 (0.67, 0.91)	0.78 (0.67, 0.92)
Sex					
Male		1.00	1.00	1.00	1.00
Female		1.05 (1.03, 1.07)	1.11 (1.08, 1.14)	1.11 (1.08, 1.14)	1.11 (1.08, 1.13)
Education status					
Primary education		1.00	1.00	1.00	1.00
Secondary education		1.25 (1.14, 1.37)	1.17 (1.09, 1.25)	1.17 (1.09, 1.25)	1.16 (1.09, 1.25)
Post‐secondary education		1.38 (1.23, 1.54)	1.26 (1.17, 1.35)	1.25 (1.17, 1.35)	1.25 (1.16, 1.35)
Employment status					
Retired		1.00	1.00	1.00	1.00
Employed		1.08 (1.02, 1.14)	1.07 (1.04, 1.09)	1.07 (1.04, 1.09)	1.06 (1.03, 1.08)
Unemployed/Others		0.93 (0.86, 1.01)	0.98 (0.92, 1.04)	0.98 (0.92, 1.04)	0.98 (0.93, 1.04)
Marital status					
Unmarried		1.00	1.00	1.00	1.00
Married		1.14 (1.09, 1.19)	1.08 (1.06, 1.09)	1.08 (1.06, 1.09)	1.07 (1.06, 1.09)
Self‐rated health					
Very good		1.47 (1.31, 1.66)	1.30 (1.20, 1.40)	1.30 (1.20, 1.40)	1.29 (1.20, 1.39)
Good		1.39 (1.24, 1.54)	1.27 (1.17, 1.37)	1.27 (1.17, 1.37)	1.26 (1.20, 1.36)
Fair		1.24 (1.16, 1.33)	1.19 (1.13, 1.26)	1.19 (1.13, 1.26)	1.19 (1.10, 1.25)
Poor		1.00	1.00	1.00	1.00
Tooth pain					
No		1.00	1.00	1.00	1.00
Yes		1.22 (1.20, 1.23)	1.24 (1.22, 1.26)	1.24 (1.22, 1.26)	1.24 (1.22, 1.26)
Income groups					
Lower Quartile		1.00	1.00	1.00	1.00
Middle‐Lower Quartile		1.12 (1.07, 1.17)	1.09 (1.05, 1.12)	1.09 (1.05, 1.12)	1.16 (1.07, 1.25)
Middle‐Upper Quartile		1.22 (1.14, 1.30)	1.13 (1.09, 1.18)	1.13 (1.09, 1.18)	1.26 (1.16, 1.36)
Upper Quartile		1.33 (1.19, 1.49)	1.20 (1.14, 1.27)	1.20 (1.14, 1.27)	1.43 (1.27, 1.61)
Current smoking status					
No		1.00	1.00	1.00	1.00
Yes		0.93 (0.89, 0.98)	0.92 (0.90, 0.95)	0.92 (0.90, 0.95)	0.92 (0.90, 0.95)
The number of Dentists		0.91 (0.85, 0.97)	0.92 (0.86, 0.97)	0.92 (0.86, 0.98)	0.92 (0.86, 0.98)
Interaction terms					
Low × Public dental expenditure					1.00
Middle‐Low × Public dental expenditure					0.995 (0.991, 0.999)
Middle‐High × Public dental expenditure					0.992 (0.988, 0.996)
High × Public dental expenditure					0.986 (0.980, 0.992)
Random intercept variance (country level)	0.25 (0.15, 0.41)		0.19 (0.11, 0.35)	0.13 (0.07, 0.22)	0.13 (0.07, 0.22)
VPC (%)	20.4		16.0	10.8	10.8
MRR	1.61 (1.45, 1.85)		1.52 (1.36, 1.75)	1.40 (1.29, 1.56)	1.40 (1.29, 1.56)

*Note:* Public dental expenditure represents the combined spending from government schemes, social health insurance schemes, and compulsory private insurance schemes. Interaction terms represent the modifying effect of income group (Low, Middle‐Low, Middle‐High, High) on the association between public dental expenditure and dental visits. Null model: Weighted (accounts for country‐level random effects). Model 1: Includes age, sex, education, employment status, marital status, self‐rated health, tooth pain, smoking status, income quartile, and number of dentists per 10 000 population. Model 2: Model 1 variables plus public dental expenditure (per $10 per capita). Model 3: Model 2 variables plus interaction terms: Public dental expenditure × income group.

In Model 3, interaction terms were included to assess whether the association between public dental expenditure and dental visits varied across income groups. Relative to the low‐income group, the interaction term was 0.995 (95% CI 0.991–0.999), 0.992 (0.988–0.996), and 0.986 (0.980–0.992) for the middle‐low‐, middle‐high‐, and high‐income groups, respectively.

As shown in Figure 2, the predicted probability of dental visits increased significantly with higher levels of public dental expenditure, particularly up to around $150 per capita. For example, among the low‐income group, the predicted probability rose from 0.29 at $0 to 0.60 at $150. A similar pattern was observed across other income groups. However, the increase at $200 was not statistically significant.

**FIGURE 2:**
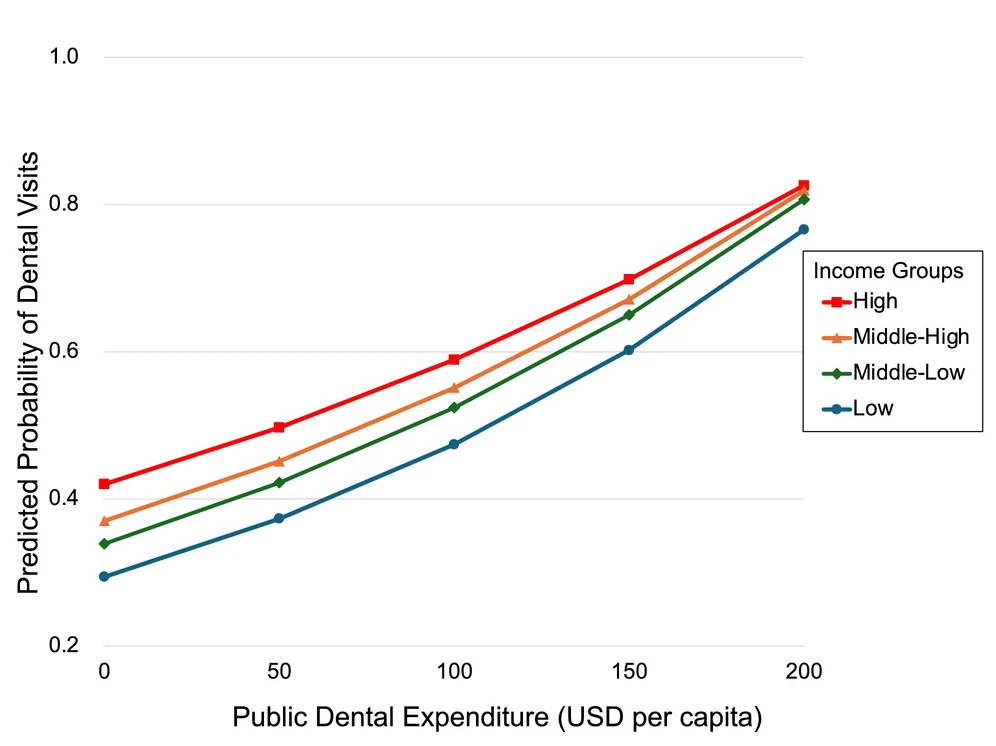
Predicted probabilities of dental visits by public dental expenditure and income group. Predicted values from Model 3. Public dental expenditure is measured in 2019 USD, adjusted for PPP.

The null model showed a VPC of 20.4%, indicating substantial between‐country variability in dental visits. Model 1, which did not include public dental expenditure, reduced the VPC to 16.0%, suggesting that individual‐level covariates partially accounted for the country‐level variance. Model 2, which additionally included public dental expenditure, further reduced the VPC to 10.8%, indicating that this variable explained an additional portion of the variance. In Model 3, which also included interaction terms between public dental expenditure and income groups, the VPC remained at 10.8%, suggesting that public dental expenditure explained a considerable portion of the between‐country variance, whereas the interaction terms did not further reduce it. Similarly, the MRR decreased from 1.61 (95% CI: 1.45–1.85) in the null model to 1.52 (95% CI: 1.36–1.75) in Model 1 and to 1.40 (95% CI: 1.29–1.56) in Model 2, and remained unchanged in Model 3, indicating a reduction in unexplained variability at the country level.

Sensitivity analyses confirmed the robustness of the main findings. Public dental expenditure remained positively associated with dental visits across all models, with prevalence ratios consistently around 1.05. The direction and magnitude of the associations, including the interaction terms with income, remained stable regardless of weighting, exclusion of Japanese participants, or inclusion of compulsory private insurance schemes. Detailed results of the sensitivity analyses are provided in Table S1 (Supporting Information).

## 4 Discussion

This study examined the association between public dental expenditure and dental visits among older adults in 25 European countries, Israel, and Japan. Higher public dental expenditure was positively associated with higher rates of dental visits. Furthermore, although dental visit rates were higher among higher‐income individuals, the association between public expenditure and dental visits was stronger among lower‐income groups, with inequalities diminishing as public spending increased. These findings highlight the role of public financial support in improving dental care access among low‐income populations.

Individual‐level barriers to dental care have been widely examined, suggesting that lower‐income individuals are more likely to forgo dental visits due to financial constraints [14, 31]. Dental insurance coverage has been identified as a key factor in reducing financial burden, thereby facilitating dental visits [16, 32]. Additionally, higher out‐of‐pocket expenses have been associated with reduced dental visits, particularly among lower‐income groups, contributing to financial hardship and unmet dental care needs [8]. These studies provide critical insights into individual‐level financial constraints. However, most cross‐national research has relied on categorical classifications, such as insurance coverage [16] or welfare regime typologies [17]. There is limited evidence assessing the association between national‐level public dental expenditure and dental visit rates. A positive association was also observed in the supplementary country‐level ecological analysis, consistent with the main multilevel findings. This study extends previous research by using public dental expenditure as a quantifiable indicator of government support, enabling direct evaluation of its impact on access to care among disadvantaged populations. In multilevel analyses, incorporating public dental expenditure explained a meaningful proportion of the cross‐national variance in dental visits. This finding highlights the role of public dental expenditure in shaping access to care at the national level. Moreover, public dental expenditure remained a significant predictor of dental visits adjusted for individual socioeconomic status, with stronger associations observed among lower‐income groups. These findings indicate the role of public financial support in mitigating socioeconomic inequalities in access to dental care and promoting equitable oral health policies. Predicted probabilities increased more gradually beyond $150 per capita, with no significant gain at $200. Since only two countries exceeded this level, future studies should include more high‐expenditure countries.

This study has several limitations. As it is cross‐sectional, causal inferences cannot be drawn. However, healthcare budgets are generally determined by long‐term policies, making reverse causality less likely. The public dental expenditure data used in this study represent total national‐level spending and were not disaggregated by age group. Therefore, the effect of public spending on older adults could not be assessed. In addition, information on oral health conditions and the need for dental services was limited, and edentulous status was not available in a comparable form across datasets. This may have affected the estimated associations, as oral health needs and reasons for dental visits may differ between dentate and edentate older adults. While the number of dentists was included as a healthcare resource indicator, the number of dental hygienists and assistants could not be included because internationally comparable data were unavailable [33]. Future research should incorporate broader measures of the dental workforce. In addition, the generalisability of the findings may be limited by the relatively small number of countries (*n* = 27). Multilevel modelling was used to account for between‐country heterogeneity, but the findings should still be interpreted with caution, given the limited number of countries.

This study provides highly relevant insights in support of the UHC framework, which emphasises equitable access to healthcare services. Japan, where most dental treatments are covered under a UHC system [13], contrasts with many European countries, where public subsidies are limited, leading to greater inequalities in dental visits [34]. Cross‐national comparison suggests a broader impact of public financial support. However, dental care remains underfunded in many public healthcare systems [35]. The findings suggest that increasing public dental expenditure may improve dental visits among lower‐income populations, supporting the need to integrate dental care into UHC policies [10].

## 5 Conclusion

This study suggests that higher public dental expenditure is associated with better access to dental services among older adults, especially among lower‐income groups. Expanding public financial support for dental care may contribute to reducing socioeconomic inequalities in dental service use, particularly in countries where dental coverage remains limited compared to general medical services. Strategic allocation of public resources may play a key role in improving equitable access to dental care within the broader UHC framework.

## Author Contributions

Yuko Inoue conceived the study, contributed to design, acquisition, analysis and interpretation, and drafted the manuscript. Jun Aida, Richard G. Watt, and Anja Heilmann contributed to design and interpretation, and critically revised the manuscript. All authors gave their final approval and agree to be accountable for all aspects of the work.

## Funding

This work was supported by JSPS KAKENHI (20H00557, 20K10540, 21H03196, 21K17302, 22H00934, 22H03299, 22K04450, 22K13558, 22K17409, 23H00449, 23H03117, 23K21500, 24KJ0065); Health Labour Sciences Research Grants (19FA1012, 19FA2001, 21FA1012, 22FA2001, 22FA1010, 22FG2001); Research Institute of Science and Technology for Society (RISTEX, JPMJOP1831, JPMJRX21K6); Japan Health Promotion and Fitness Foundation; Futokukai Foundation; and TMDU priority research area grants. This study used data from SHARE Wave 8 and JAGES (the Japan Gerontological Evaluation Study). The SHARE data collection has been funded by the European Commission through various Framework Programmes and by additional national and international sources (see www.share‐eric.eu for the full list). The views and opinions expressed in this article are those of the authors and do not necessarily reflect the official policy or position of the respective funding organisations.

## Conflicts of Interest

The authors declare no conflicts of interest.

## Supporting information


**Table S1:** Sensitivity Analysis: Multilevel Modified Poisson Regression of Dental Visits.
**Figure S1:** Country‐level ecological analysis of the association between public dental expenditure per capita and dental visit rates among older adults.

## Data Availability

The data that support the findings of this study are available from SHARE‐ERIC and JAGES under data use agreements. Access to SHARE data requires registration with the SHARE Research Data Centre (https://share‐eric.eu/data/data‐access) and compliance with the SHARE Conditions of Use. Access to JAGES data requires approval from the JAGES project team and is subject to application and data use agreements as detailed on the JAGES website (https://www.jages.net/kenkyuseika/datariyou/). The authors are not permitted to share these data directly.
