# Supplementary material for: Public Dental Expenditure and Dental Visits in Europe and Japan
Source: Community Dent Oral Epidemiol. 2026 May 31;54(5):578–87. doi: 10.1111/cdoe.70076 (PMC13669807; doi:10.1111/cdoe.70076)
Supplement: Supplementary file 1 [file CDOE-54-578-s001.docx]

Table S1. Sensitivity Analysis: Multilevel Modified Poisson Regression of Dental Visits

|  | **Model 3A** | **Model 3B** | **Model 3C** |
| --- | --- | --- | --- |
|  | PR (95% CI) | PR (95% CI) | PR (95% CI) |
| Public dental expenditure 　　　　　　　　(unit = 10 USD per capita) | 1.05 (1.03–1.07) | 1.05 (1.03–1.08) |  |
| Government and Social health insurance dental expenditure (unit =10 USD per capita) |  |  | 1.05 (1.03–1.08) |
| Age (years) |  |  |  |
| 65-74 | 1.00 | 1.00 | 1.00 |
| 75-84 | 0.97 (0.88–1.07) | 0.89 (0.84–0.94) | 0.96 (0.87–1.05) |
| ≥ 85 | 0.79 (0.68–0.91) | 0.70 (0.63–0.78) | 0.78 (0.67–0.92) |
| Sex |  |  |  |
| Male | 1.00 | 1.00 | 1.00 |
| Female | 1.11 (1.09–1.13) | 1.12 (1.09–1.16) | 1.11 (1.08–1.14) |
| Education status |  |  |  |
| Primary education | 1.00 | 1.00 | 1.00 |
| Secondary education | 1.16 (1.09–1.22) | 1.21 (1.14–1.29) | 1.16 (1.09–1.25) |
| Post-secondary education | 1.25 (1.17–1.33) | 1.31 (1.21–1.42) | 1.25 (1.16–1.35) |
| Employment status |  |  |  |
| Retired | 1.00 | 1.00 | 1.00 |
| Employed | 1.06 (1.04–1.07) | 1.01 (0.96–1.07) | 1.06 (1.03–1.08) |
| Unemployed/ Others | 0.99 (0.94–1.03) | 0.96 (0.88–1.04) | 0.98 (0.93–1.04) |
| Marital status |  |  |  |
| Unmarried | 1.00 | 1.00 | 1.00 |
| Married | 1.07 (1.05–1.08) | 1.07 (1.05–1.10) | 1.07 (1.06–1.09) |
| Self-rated health |  |  |  |
| Very good | 1.28 (1.20–1.37) | 1.27 (1.19–1.36) | 1.29 (1.20–1.39) |
| Good | 1.26 (1.17–1.34) | 1.26 (1.17–1.36) | 1.26 (1.17–1.36) |
| Fair | 1.19 (1.13–1.25) | 1.18 (1.12–1.25) | 1.19 (1.12–1.25) |
| Poor | 1.00 | 1.00 | 1.00 |
| Tooth pain |  |  |  |
| No | 1.00 | 1.00 | 1.00 |
| Yes | 1.20 (1.19–1.22) | 1.31 (1.17–1.47) | 1.24 (1.22–1.26) |
| Income groups |  |  |  |
| Lower Quartile | 1.00 | 1.00 | 1.00 |
| Middle-Lower Quartile | 1.14 (1.06–1.22) | 1.15 (1.07–1.23) | 1.16 (1.08–1.24) |
| Middle-Upper Quartile | 1.25 (1.16–1.35) | 1.23 (1.15–1.31) | 1.27 (1.17–1.37) |
| Upper Quartile | 1.40 (1.25–1.57) | 1.39 (1.25–1.54) | 1.43 (1.27–1.61) |
| Current smoking status |  |  |  |
| No | 1.00 | 1.00 | 1.00 |
| Yes | 0.91 (0.88–0.95) | 0.93 (0.91–0.96) | 0.92 (0.90–0.95) |
| The number of dentists | 0.92 (0.87–0.99) | 0.92 (0.86–0.98) | 0.91 (0.86–0.97) |
| Interaction terms |  |  |  |
| Low × Public dental expenditure | 1.00 | 1.00 | 1.00 |
| Middle-Low × Public dental expenditure | 0.996 (0.993–0.999) | 0.995 (0.991–1.000) | 0.995 (0.991–0.999) |
| Middle-High × Public dental expenditure | 0.992 (0.989–0.996) | 0.993 (0.989–0.998) | 0.991 (0.987–0.995) |
| High × Public dental expenditure | 0.987 (0.982–0.993) | 0.987 (0.979–0.995) | 0.985 (0.979–0.992) |
|  |  |  |  |
| Random intercept variance (country level) | 0.14 (0.08–0.24) | 0.13 (0.07–0.22) | 0.13 (0.07–0.22) |
| VPC(%) | 11.8 | 10.8 | 10.8 |
| MRR | 1.42 (1.31–1.59) | 1.40 (1.29–1.56) | 1.40 (1.29–1.56) |

Note:

Public dental expenditure represents the combined spending from government schemes, social health insurance schemes, and compulsory private insurance schemes.

Interaction terms represent the modifying effect of income group (Low, Middle-Low, Middle-High, High) on the association between public dental expenditure and dental visits.

Model 3A: Unweighted version of the main analysis model (Model 3 in the main text).

Model 3B: Same as Model 3 but limited to the SHARE dataset (excluding participants from Japan).

Model 3C: Same as Model 3 but excludes compulsory private insurance expenditure, including only government and social health insurance schemes.

Figure S1. Country-level ecological analysis of the association between public dental expenditure per capita and dental visit rates among older adults

Note:

Each point represents one country. A positive association was observed (Pearson’s r = 0.61, p = 0.0008; Spearman’s rho = 0.60, p = 0.0009). In linear regression, each 10 USD per capita increase in public dental expenditure was associated with a 2.19 percentage-point higher dental visit rate.
